# Idiopathic Gingival Fibromatosis: Report of a Rare Case

**DOI:** 10.7759/cureus.67448

**Published:** 2024-08-21

**Authors:** N.S. Shree Abiraami, T.N. Umamaheswari, Karthikeyan Ramalingam, Devika S Pillai

**Affiliations:** 1 Oral Medicine and Radiology, Saveetha Dental College and Hospitals, Saveetha Institute of Medical and Technical Sciences, Saveetha University, Chennai, IND; 2 Oral Medicine and Radiology, Oral Medicine and Radiology, Saveetha Dental College and Hospitals, Saveetha Institute of Medical and Technical Sciences, Saveetha University, Chennai, IND; 3 Oral Pathology and Microbiology, Saveetha Dental College and Hospitals, Saveetha Institute of Medical and Technical Sciences, Saveetha University, Chennai, IND

**Keywords:** gingival overgrowth, gingival hyperplasia, idiopathic gingival fibromatosis, gingivectomy, gingival fibromatosis, gingival enlargement

## Abstract

The progressive overgrowth of the gingiva is the hallmark of idiopathic gingival fibromatosis (IGF). Excess gingival tissue can obscure the crown of a tooth, resulting in spaces between teeth, displacement, retention of primary or permanent teeth, and difficulties with feeding, speaking, and appearance. The diagnosis and management of inherited gingival fibromatosis are the focus of this case report. A 12-year-old girl was referred from the Department of Orthodontics to Oral Medicine as a result of progressive gingival enlargement, which impeded orthodontic treatment for misaligned lower front teeth. The patient underwent a conservative periodontal treatment regimen that encompassed gingivectomy and debridement. The excised gingival tissues were submitted for histopathological examination. Tissue sections stained with hematoxylin and eosin showed connective tissue with dense bundles of collagen fibers and little inflammation. The patient was reviewed after three months, and advised of orthodontic management for further aesthetic correction. The findings indicated that the oral symptoms of gingival fibromatosis are influenced by the severity of the condition and the age at which it begins. Early intervention helps mitigate potential difficulties for younger individuals.

## Introduction

A rare and largely unknown disorder known as idiopathic gingival fibromatosis (IGF) is characterized by the gradual and frequent symmetrical proliferation of gingival tissue. As a result of this illness, affected individuals may experience serious difficulties with their overall quality of life and oral health [1]. IGF is unique in that its exact source is unknown, and it is not connected to any recognized genetic abnormality or systemic disease, unlike gingival hyperplasia, which is linked to certain systemic disorders or a side effect of specific drugs [2].

IGF can occur at any age; however, it usually shows up in childhood or adolescence [3]. Due to the condition's degenerative nature, the gingival tissue progressively enlarges, frequently resulting in a noticeable increase in volume. This increase in size could potentially hinder the ability to communicate, masticate, and maintain optimal oral hygiene [4]. As the disease worsens, the enlarged gingival tissue can make dental operations difficult, extremely uncomfortable, and adversely affect the patient’s appearance when they smile. The gingiva is fibrotic, firm to hard in texture, and its pale appearance further accentuates the clinical manifestations of the disease.

IGF pathophysiology is poorly understood, and little is known about the condition's underlying processes [5]. Because it is not associated with any specific genetic abnormalities or systemic diseases, the syndrome lacks a clear etiology. We categorize it as idiopathic because we have not found any obvious cause, and there is no known connection to drugs or other external variables that frequently induce gingival overgrowth. To diagnose IGF, a comprehensive clinical exam is necessary. We might also do a histological analysis to rule out other possible reasons for the gingival overgrowth, such as drug-induced gingival hyperplasia or systemic disease-induced gingival enlargement [6]. To manage symptoms and stop recurrence, treatment usually consists of supportive dental care in addition to surgical procedures to remove excess tissue. Routine monitoring is necessary to address any potential issues and ensure the best possible dental health.

To address the condition's wider effects, the management of idiopathic gingival fibromatosis necessitates a multidisciplinary approach involving not just dental experts but also potentially other healthcare practitioners. Sustained investigation is required to advance knowledge of IGF, increase diagnostic techniques, and create more effective treatment plans that will improve the quality of patient care for those afflicted with this challenging illness [7].

## Case presentation

**Figure 1:**
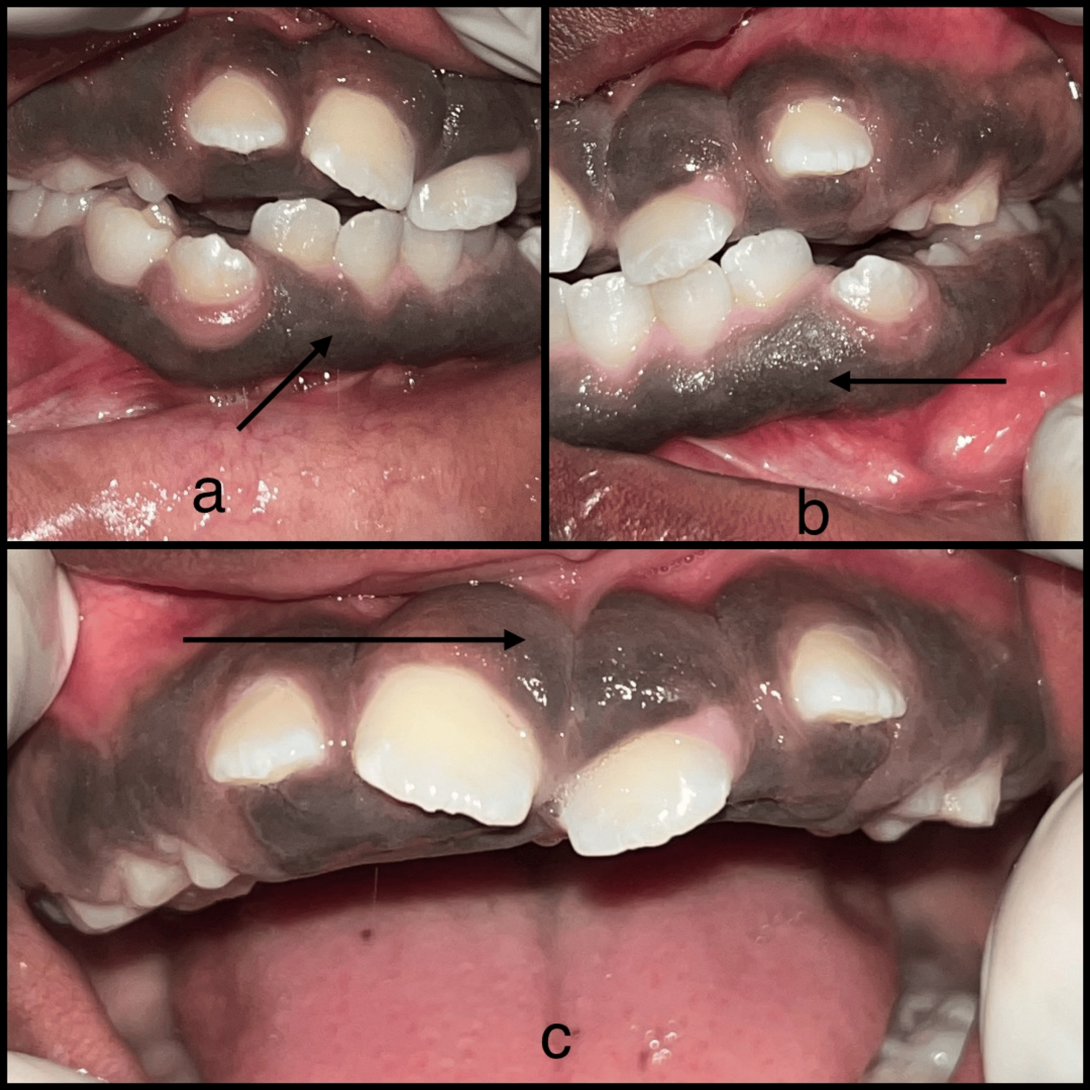
Intraoral images Figures 1a, 1b, 1c show pigmented, blunt-edged gingiva.

A 12-year-old female patient presented to the Department of Oral Medicine and Radiology at Saveetha Dental College and Hospital in Chennai, Tamil Nadu, India, with the major complaint of poor gingival aesthetics, incompetent lips, and gaps between the teeth. The patient stated that the gingival growth began when their permanent teeth came in and gradually spread to encompass all of their permanent teeth. The patient did not disclose any history of drug use, mental disorders, or hormonal changes that could be associated with gingival alterations. The patient's parents did not disclose any signs of gingival overgrowth. During the intraoral examination, the attached gingiva was found to be swollen and pigmented all over, almost covering the coronal third of the teeth and this led to the loss of stippling (Figure 1a, 1b, 1c).

On palpation, all inspectory findings were confirmed. The growth was firm and fibrous in consistency with no tenderness.

**Figure 2:**
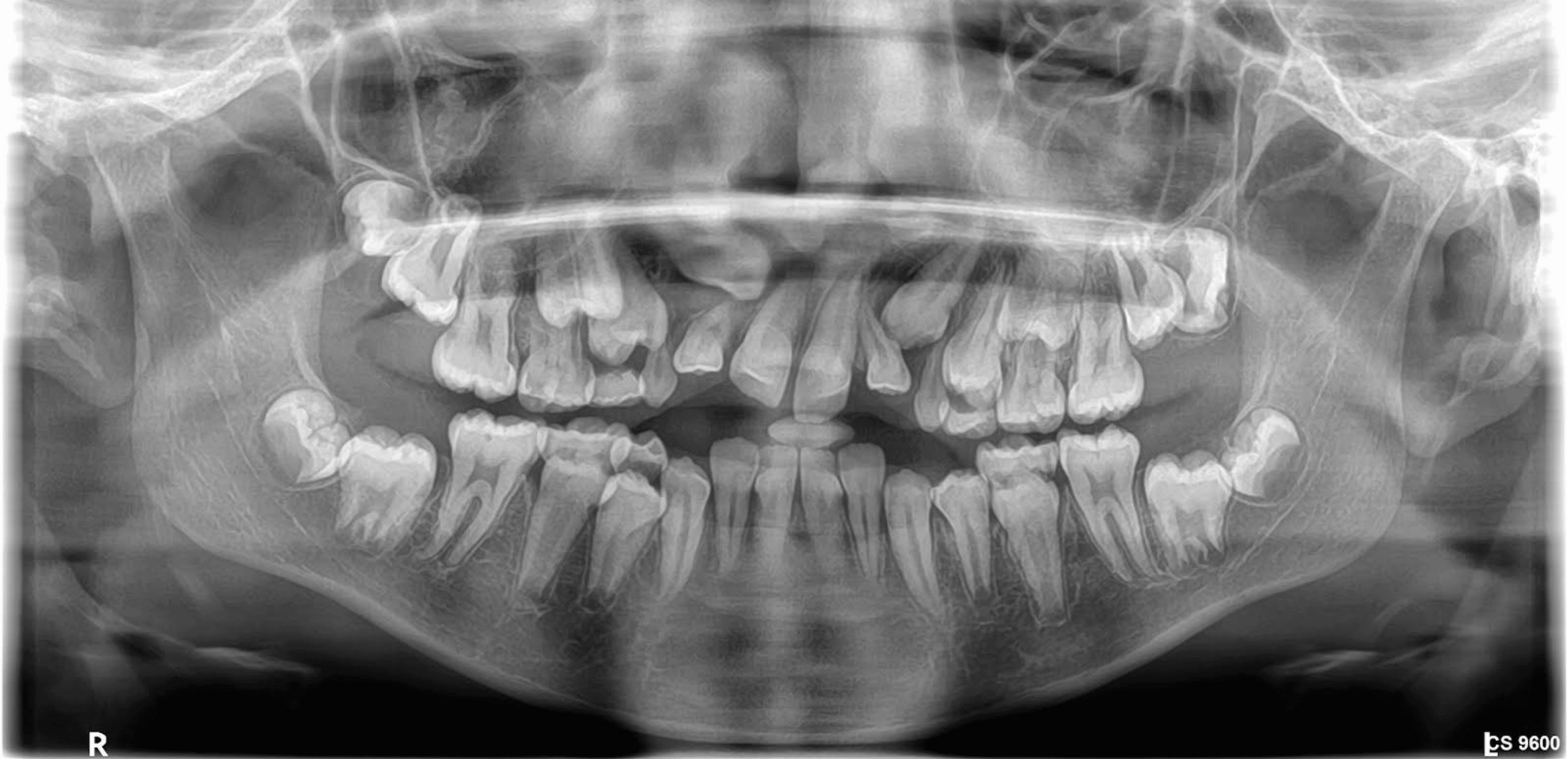
Orthopantomographic image Figure 2 shows an orthopantomogram with multiple exfoliating areas at 54, 53, 63, 75, 84, and 85, as well as erupting areas at 14, 15, 12, 23, 24, 25, 35, 45, and 44.

**Figure 3:**
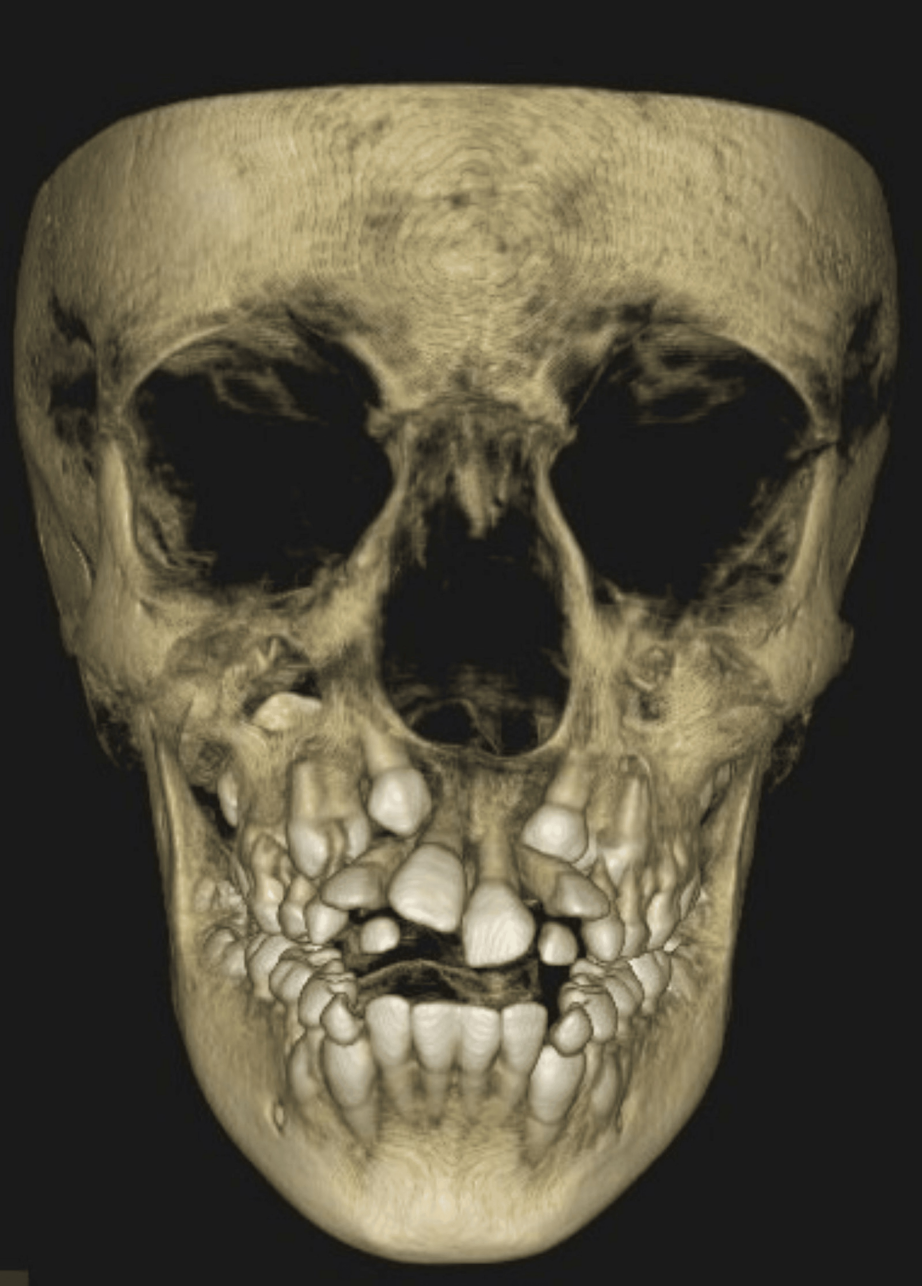
Full skull CBCT A full-skull CBCT shows mixed dentition, developing permanent tooth buds evident, and exfoliating 53, 54, 63, 75, 83, 84, and 85. CBCT: Cone beam computed tomography

With the aim of ruling out fibro-osseous lesions, radiographic and lab investigations were done. Radiographic imaging revealed the multiple impacted and retained deciduous teeth (Figures 2, 3). 

**Figure 4:**
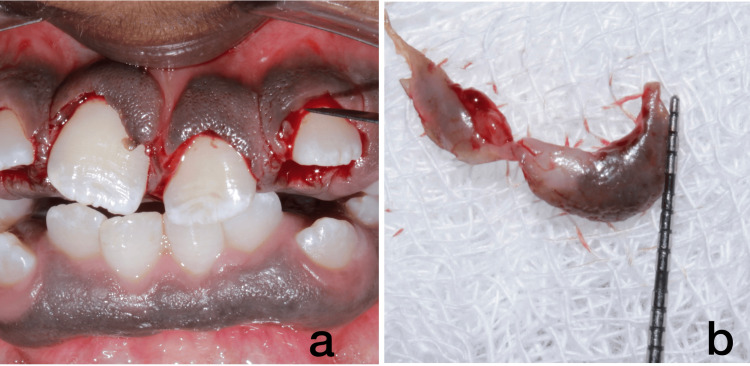
Gingivectomy in Upper Anterior 4a and 4b depict scalpel surgery done in relation to regions 11, 21, and 22.

The patient's blood samples were collected to assess serum calcium, alkaline phosphatase, and thyroid levels. All the tests were within the physiological limits of T3- 165 ng/dL, T4- 7 µq/dL, TSH- 1.19 uIU/mL, alkaline phosphatase- 193 U/L, and serum calcium- 9 mg/dL. As it is we did a gingivectomy and an excisional biopsy. The histological examination showed that the connective tissue had gotten bigger, but there were no blood vessels, and the collagen fibers were packed together tightly (Figure 4a, 4b). We obtained informed consent by discussing the treatment with the patient, taking into account their requirements and the severity of the enlargement.

**Figure 5:**
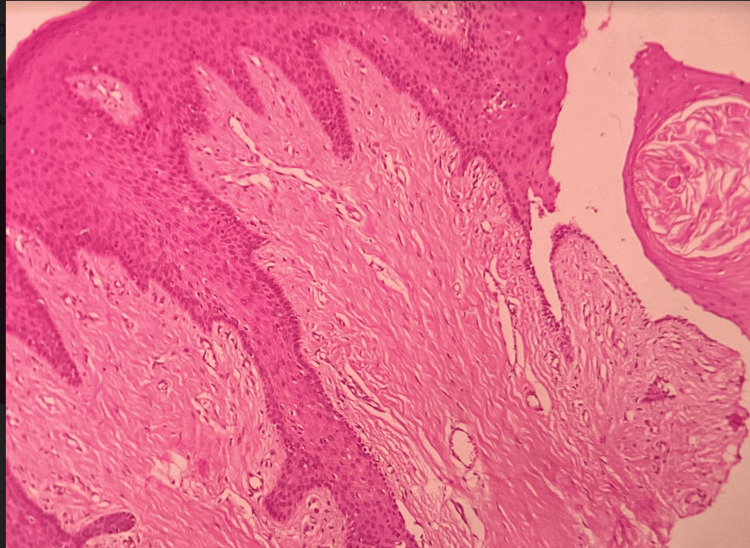
Histopathological picture of the tissue sample Figure 5 depicts histopathology picture showing dense fibrous connective tissue stroma with minimal inflammation along with surface epithelium (H&E, 10x).

Additionally, there were numerous fibroblasts and a slight presence of chronic inflammatory cells, predominantly lymphocytes, and plasma cells, numerous endothelial-lined capillary spaces, and extravasated RBCs. The surface epithelium showed hyperplasia and had extended rete ridges, indicating a histological diagnosis of fibro-epithelial hyperplasia (Figure 5).

**Figure 6:**
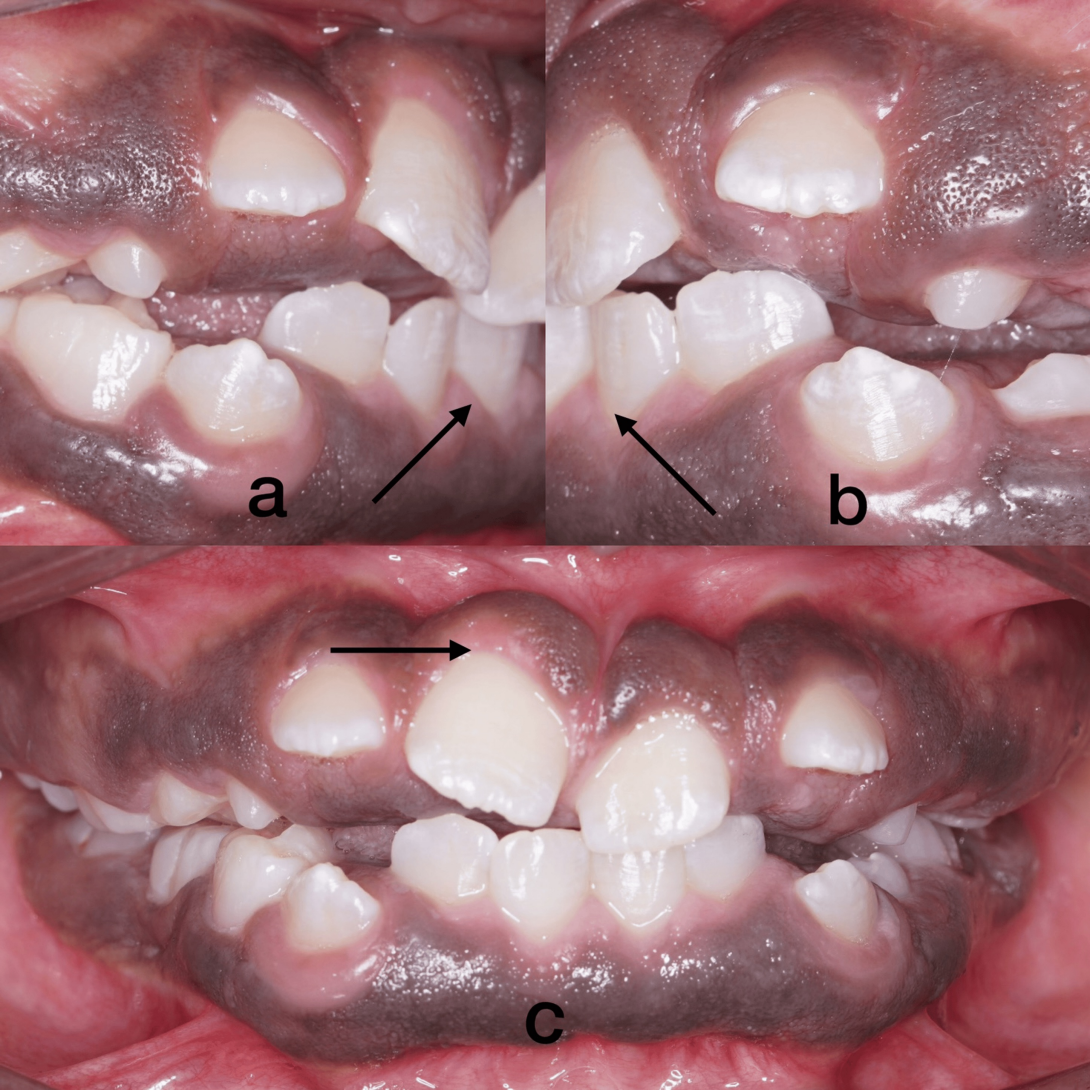
Post-treatment follow-up images. Figures 6a, 6b, 6c show a more defined knife-edge margin of the gingiva post-gingivectomy. The teeth are more pronounced after gingivectomy.

The diagnosis of IGF (idiopathic gingival fibromatosis) was based on the individual's medical, family, and medication history, as well as the clinicopathological correlation. We obtained informed consent for gingivectomy by discussing the treatment with the patient, taking into account their requirements and the severity of the enlargement. Administered a local anesthetic using 2% lignocaine and a 1:80,000 epinephrine ratio, we used a scalpel to precisely remove any excess gingival tissue during the procedure. Following the upper anterior gingivectomy procedure, the patient was prescribed antibiotics and analgesics for post-treatment pain management. They also received advice to use 10 ml of chlorhexidine mouth rinses twice daily for 10 days. The patient's recovery after the surgery went without any complications, and they were content with the therapy they received. The patient undergoes a routine check-up every three months (Figure 6a, 6b, 6c). We referred the patient back to the orthodontist for further management of tooth alignment and aesthetic concerns. 

## Discussion

Idiopathic Gingival Fibromatosis (IGF) is a rare, benign oral condition characterized by the overgrowth of gingival tissue. Despite its benign nature, IGF can cause significant functional and aesthetic challenges, impacting patients' quality of life. The exact etiology of IGF remains elusive, presenting a considerable challenge in developing targeted treatments [8].

The precise cause of IGF is not well understood, complicating efforts to develop effective interventions. Current research suggests a potential hereditary component, with both autosomal dominant and autosomal recessive patterns reported in familial cases [9][10]. Despite these findings, the specific genetic mechanisms underlying IGF remain largely unknown, necessitating further research to elucidate these pathways. Genetic factors play a crucial role in the inheritance and manifestation of certain conditions, such as idiopathic gingival fibromatosis (IGF). IGF can be inherited in an autosomal dominant pattern, requiring only one copy of the altered gene to cause the condition [11]. Conversely, it can also follow an autosomal recessive pattern, necessitating two copies of the altered gene-one from each parent. When specific genetic mutations, such as those in the Son-of-Sevenless gene (SOS1) on chromosome 2p21, occur, nonsyndromic IGF impacts cellular signal transduction. Additionally, genetic alterations in Type I collagen metabolism are known to contribute to abnormal cell proliferation and gingival overgrowth (Table 1).

**Table 1:** Association of genetic factors with IGF prevalence. IGF: Idiopathic Gingival Fibromatosis

Genetic Factors	Description
Autosomal Dominant Inheritance	IGF can be inherited in an autosomal dominant pattern, meaning only one copy of the altered gene is needed to cause the condition.
Autosomal Recessive Inheritance	IGF can also be inherited in an autosomal recessive pattern, requiring two copies of the altered gene (one from each parent) to cause the condition.
Son-of-Sevenless Gene (SOS1) Mutation	Mutations in the SOS1 gene on chromosome 2p21 are associated with non-syndromic IGF, affecting cellular signal transduction.
Type I Collagen Alterations	Genetic alterations in collagen metabolism, particularly Type I collagen, contribute to abnormal cell proliferation and gingival overgrowth.

Excessive gingival tissue growth, which can interfere with oral functions like chewing and speaking and cause significant aesthetic concerns that may lower self-esteem, is the clinical hallmark of IGF. The condition typically affects all teeth, with a generalized-to-localized ratio of 15.2:1. It's important to make a correct diagnosis because some symptoms are similar to those of other conditions, like gingival gigantism, elephantiasis gingiva, fibromatosis gingivae, congenital macro gingiva, hereditary gingival hyperplasia, and hypertrophic gingiva. Recent advances in machine learning have improved the early detection of lesions [12]. A correct diagnosis is essential for accurate management and treatment planning. In this case, the initial investigation with orthopantomography revealed multiple impacted and retained deciduous teeth. Further cone beam computed tomography (CBCT) imaging was performed to rule out any associated syndromes and proper treatment planning of the impacted tooth. Furthermore, IGF could be linked to bone resorption or deformities caused by long-term inflammation or pressure exerted by the enlarged gingiva. CBCT has the ability to accurately identify these alterations.

Idiopathic gingival fibromatosis (IGF) is a rare condition that causes non-inflammatory gingiva enlargement due to excessive connective tissue growth. The prevalence of idiopathic gingival fibromatosis is quite low, estimated at 1 in 175,000 individuals. This condition can significantly impact oral function and aesthetics, often requiring surgical intervention to manage the overgrowth and maintain oral health [13].

IGF management depends on the severity of the gingival enlargement. In mild cases, rigorous oral hygiene and regular dental visits may suffice to control the condition. However, severe cases often require surgical intervention to remove the excess tissue and restore function and appearance [14]. Gingivectomy and gingivoplasty are two common surgical procedures for severe enlargement without attachment loss. Techniques such as conventional external bevel gingivectomy, electrocautery, and diode laser application are employed, with a preference for diode lasers due to their hemostatic properties, patient comfort, reduced healing time, and lower morbidity. Nonetheless, diode laser use is not without limitations, including risks of lateral heat damage, depth control challenges, technical skill requirements, and higher costs. The differential diagnosis of idiopathic gingival fibromatosis is hereditary gingival fibromatosis (HGF), drug-induced gingival enlargement, leukemia-associated gingival hyperplasia, vitamin C deficiency (scurvy), fibrotic hyperplasia, sarcoidosis, orofacial, and orofacial granulomatosis (Table 2).

**Table 2:** Differential diagnoses for idiopathic gingival fibromatosis

Condition	Characteristics
Hereditary Gingival Fibromatosis (HGF)	Slow progressive gingival enlargement, often hereditary, with firm and pale pink gingiva.
Drug-Induced Gingival Enlargement	Gingival overgrowth caused by medications such as phenytoin, cyclosporine, and calcium channel blockers, is typically associated with poor oral hygiene.
Leukaemia-associated Gingival Hyperplasia	Gingival enlargement due to infiltration of leukemic cells, is often accompanied by bleeding, bruising, and general malaise.
Vitamin C Deficiency (Scurvy)	Gingival swelling and bleeding, often with other signs of vitamin deficiency such as fatigue and skin changes.
Fibrous Hyperplasia	Localized overgrowth due to chronic irritation, usually at sites of trauma or irritation
Sarcoidosis	A chronic inflammatory disease can cause gingival enlargement along with systemic signs like lymphadenopathy and lung involvement.
Orofacial Granulomatosis	Chronic granulomatous inflammation leads to gingival swelling, often with lip swelling and other oral lesions.

The presented case study observed IGF in siblings across multiple generations, underscoring the hereditary nature of the disease [15]. Genetic testing facilitated the construction of a family tree spanning four generations, underscoring the importance of genetic counseling and family history in managing IGF. The treatment involved diode laser therapy, which proved effective in avoiding more invasive surgical interventions. This case emphasizes the need for personalized treatment approaches based on genetic insights and the potential benefits of minimally invasive techniques. Patient education is a critical component of IGF management. We should inform patients about the importance of maintaining oral hygiene, the potential for disease recurrence, and the likelihood of the condition affecting future generations [16]. Regular dental check-ups are essential for monitoring and managing any recurrence of gingival overgrowth [17]. IGF presents significant challenges due to its unclear etiology and complex clinical manifestations. Advances in genetic research and minimally invasive treatment options offer hope for better management of this condition [18]. In the future, continued research will be critical to fully understanding the genetic factors involved and developing more effective, targeted therapies.

## Conclusions

Idiopathic gingival fibromatosis is an uncommon illness with a poorly understood cause and a low rate of recurrence. Effective management of idiopathic gingival fibromatosis is critical because it can lead to significant cosmetic and psychological issues in patients, including difficulties chewing, speaking, and tooth misalignment. Although we cannot predict recurrence, the psychological and functional benefits greatly outweigh the risk. The patient demonstrated non-syndromic idiopathic gingival hypertrophy. A complete gingival hypertrophy excision improved aesthetics and mastication.
